# Attention Deficit Hyperactivity Disorder in Girls and the Risk of Unplanned Pregnancies

**DOI:** 10.3390/children12010062

**Published:** 2025-01-07

**Authors:** Florina Rad, Alexandra Mariana Buică, Nicolae Gică

**Affiliations:** 1 Child and Adolescent Psychiatry Department, Carol Davila University of Medicine and Pharmacy, 020021 Bucharest, Romania; florina.rad@umfcd.ro; 2 Child and Adolescent Psychiatry Department, “Prof. Dr. Al. Obregia” Clinical Hospital of Psychiatry, 041914 Bucharest, Romania; 3 Department of Obstetrics and Gynecology, Carol Davila University of Medicine and Pharmacy, 020021 Bucharest, Romania; gica.nicolae@umfcd.ro; 4 Filantropia Clinical Hospital, 011171 Bucharest, Romania

**Keywords:** ADHD, girls, pregnancy, comorbidity, obstetrical complications

## Abstract

Background/Objectives: Although ADHD in adults has become visible and inclusive in recent years in diagnostic manuals, research is still limited regarding the long-term outcomes of patients with this disorder. The main objective of this research was to address the many facets of predictor variables in girls with ADHD facing unplanned pregnancies at young ages in order to improve the management of pre-, peri-, and postnatal complications that may occur, as well as for early psychiatric diagnosis and effective intervention. Methods: PubMed and Web of Science Databases were used to perform literature research, and a total of 27 records were selected and used for data extraction. Results: Related articles have included the persistence of ADHD symptoms comorbid with other disorders among girls with ADHD as a risk factor for teenage pregnancies. Conduct disorders and substance use disorders are the main co-occurrent diagnoses that increase the likelihood for teenage childbirth. Unplanned pregnancies at young ages are associated with unfavorable psychosocial trajectories both for the mother and the child. Conclusions: In this review, we emphasize the importance of predisposing factors for risky sexual behaviors and unplanned pregnancies in cases of girls with ADHD. The topic of the article draws attention to the need for uniform national individualized care of girls with ADHD, the implementation of programs to prevent academic failure and early parenthood as well as addressing comorbid obstetrical and psychiatric conditions, especially in regions where the rate of adolescent births continues to be high.

## 1. Introduction

Attention Deficit Hyperactivity Disorder (ADHD) is a neurodevelopmental disorder frequently diagnosed among pediatric patients, but researchers and clinicians have paid more attention in recent years to symptoms characteristic in adulthood, which are much more difficult to recognize than in the case of children or adolescents due to their polymorphous nature. For a long time, the diagnostic criteria were oriented towards ADHD in children, but the clinical picture manifests differently in adulthood, reflecting the changes in daily activities and responsibilities characteristic of adulthood.

ADHD is characterized by an early onset, and by a combination of hyperactivity, lack of attention, inability to correctly use achieved skills, restlessness, impulsivity, and a high degree of distractibility [1]. These particularities are persistent over time. Recent research demonstrated that in some patients there is a reduction in the severity of symptoms (generally hyperactivity), but in 65% of them the symptoms remain distressing, with 90% of them experiencing dysfunction in adulthood [2]. Although the diagnostic criteria in *Diagnostic and Statistical Manual of Mental Disorders* (DSM-5) have been modified to be more inclusive of adult symptoms, this disorder is still underdiagnosed and significantly undertreated.

During the transition to adolescence, there are concerns regarding the clinical evolution of a patient with ADHD due to low inhibitory control and poor planning skills. Deficits in emotional self-regulation and at the level of executive functions become exacerbated in adulthood [3]. Although these deficits are not criteria exclusively applicable to patients with ADHD, they are characteristic of this pathology, and their presence is correlated with engagement in risky behaviors that predict negative outcomes in multiple areas of life. Risky sexual behaviors in the case of adolescent girls with ADHD consist of first sexual intercourse at young age, inconstant use of protection, increased risk for sexually transmitted diseases, multiple partners, and unplanned pregnancies before the age of consent [4].

Being one of the most diagnosed mental health disorders of childhood, ADHD is reported to affect 3% to 10% of school-age children, 2–6% of adolescents, and 4–5% in adults [5]. ADHD is diagnosed more frequently among boys than among girls, with the ratio reported in the specialized literature being 2:1, and this proportion tends to equalize in adults [6]. By age group, the prevalence of ADHD is higher among schoolboys than among male adolescents, while the prevalence among girls is lower but more stable over time [7]. Even if the tendency of girls to develop externalizing and rule-breaking behaviors is lower than in the case of boys, the studies that compared groups of teenage girls with ADHD with teenage girls without ADHD demonstrated worse outcomes in education, social relationships, and academic performances for the former [8].

Long-term impairments in young girls need further investigation in future research, since the symptoms of ADHD in adults have received increased attention in recent years with the modification of the diagnostic criteria. Even though the influence of ADHD in early involvement in a pregnancy has been demonstrated, there is still a great need to explore the underlying mechanisms and childhood predictors for impulsive sexual behaviors and failure to used contraception at a young age.

## 2. Methods

The core objective of the current research was to highlight the knowledge regarding the underlying mechanisms that increase the likelihood for teenage girls with ADHD to be involved in early pregnancies. PubMed and Web of Science Databases were used to perform a thorough literature search for this review. To guarantee a comprehensive analysis of the pertinent literature, we used a variety of keywords like “ADHD”, “ADHD in girls”, “ADHD in teenagers”, “ADHD and risk for pregnancy”, “ADHD comorbidity”, “obstetrical complications in ADHD or young mothers”, “teenage pregnancy and outcome”, and “mental health”, “risk factors for early pregnancies”, “interventional programmes for unplanned pregnancies”, “psychosocial interventions”, “maternal ADHD”, as well as using “Attention Deficit” and “Hyperkinetic Disorder” instead of ADHD/ADD. The methodology for selecting the articles was performed using the Preferred Reporting Items for Systematic Reviews and Meta-Analysis (PRISMA) statement [9].

The selected records were individually analyzed to meet the inclusion criteria of the search in the databases. We selected articles that exposed mediating factors for unplanned pregnancies in girls with ADHD, predictors for risky sexual behaviors in patients with ADHD, risk factors for negative longitudinal trajectory in this category of patients, birth outcomes and obstetrical complications associated with teenage pregnancy, psychosocial intervention for ADHD adolescent parents, and educational programs and current perspectives for teen pregnancy prevention. To ensure that the selected materials were understood and correctly analyzed, we only included reports that were exclusively written in English. Exclusion criteria were as follows: animal or in vitro studies, studies of target groups of patients not including adolescents, case reports, and case series.

**Figure 1:**
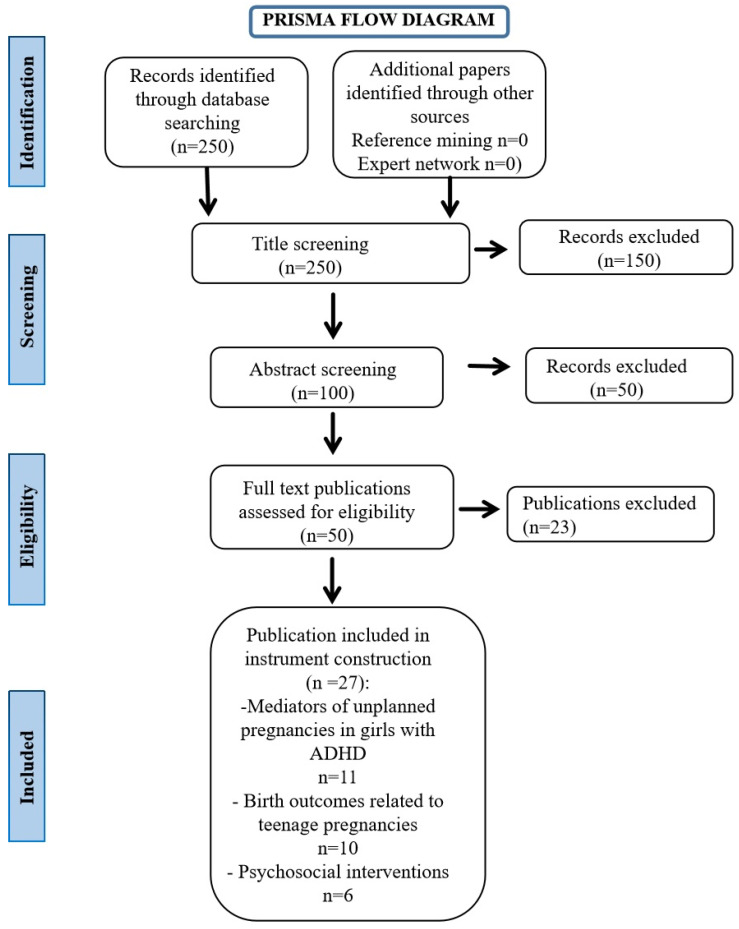
PRISMA flow diagram.

Our search parameters generated 250 records in total (Figure 1). A total of 150 papers were eliminated after the titles were screened and duplicates were eliminated. In the screening processes, we excluded 73 papers due to the following: unrelated topics, not published in English, repeated papers, case reports, case series, animal studies, no abstract available, and study protocols not available. In the last phase of the paper inclusion process, 27 studies were selected, fully analyzed, and separated according to the three major factors we aimed to examine: mediators of unplanned pregnancies in girls with ADHD, birth outcomes related to teenage pregnancies, and psychosocial interventions for this category of patients.

The goal of the search was to find a large number of papers that addressed the many facets of predictor variables in girls with ADHD facing unplanned pregnancies at young ages, in order to improve the management of pre-, peri-, and postnatal complications that may occur, as well as for early psychiatric diagnosis and effective intervention. There is still a lack of research addressing sex differences in symptom evolution in ADHD patients, with most specialized studies focusing on male dominated samples. Because girls predominantly present the silent form of ADHD with attention deficit and because the prevalence of this disorder is lower than in boys at younger ages, female presentation has been overlooked in past research.

## 3. Mediating Factors in the Occurrence of Unplanned Pregnancies in Girls with ADHD

More than half of the pregnancies worldwide are unintended or unplanned [10]. The young age of the mother at conception and the presence of mental health disorders or abuse of psychoactive substances are among the most incriminated risk factors for unplanned or unwanted pregnancies [10].

Until now, teenage pregnancies in ADHD girls have not been complexly explored in research data, because the majority of the studies investigating this topic have relied on self-measuring instruments. Such investigations do not allow generalization to larger cohorts. The update of the diagnostic criteria applicable to adults in DSM-5 has recently brought the evolution of ADHD in young adults to the attention of researchers. The problem of unplanned pregnancies in young women and teenagers with ADHD brings about psychosocial concerns such as additional mental health disorders for the mother, child maltreatment and developmental disorders, or the need for social assistance.

The rate of unplanned pregnancies in girls with ADHD compared to those without ADHD is reported by studies as varying between 42 and 45% [10]. Also, in the case of boys, the presence of this diagnosis is associated with an increased probability of early parenthood (25%) [11]. Starting from these worrying statistics, we consider the knowledge and research of the factors that could mediate the relationship between persistent ADHD symptoms and early pregnancy of great importance.

ADHD is described as a disorder of executive functions, such as response inhibition, working memory, or deficits in executive control. Research on the neuropsychology of ADHD children found a pattern of cognitive deficiencies associated with prefrontal executive function impairments: inattention, difficulties in self-regulation, deficiencies in inhibitory responses, restlessness or hyperactivity, and in certain situations, lack of interest. Executive functions reported to be deficient in ADHD patients are response inhibition, vigilance, working memory, and planning [12]. The same authors suggest that ADHD patients have difficulty delaying gratification, preferring small, immediate rewards over delayed but more consistent ones. Considering the deficits in organization, planning, flexibility, inhibition, and control of motor actions that characterize executive functions, it is possible to explain the impulsiveness of girls with ADHD in engaging in risky sexual behaviors, neglecting the use of contraceptives and experimenting with multiple sexual partners.

In 75% of cases, ADHD co-occurs with one or more psychiatric illnesses. Three concurrent medical conditions are typically present in ADHD patients [13]. This indicates that ADHD is not a minor ailment, but rather a disorder that is frequently misdiagnosed in individuals who have already sought treatment for more complicated issues that may not respond to therapy. Thus, it can be concluded that ADHD in patients whose symptomatology does not remit or is not treated in time leads to the chronicity of comorbid disorders. The presence of one or more mental health disorders is often the rule and not the exception for adolescents with ADHD. Children with the most severe disruptive behaviors generally present with the coexistence of ADHD and conduct disorder, with the comorbidity prevalence reported to be between 35% and 50% in these children [14]. They show the onset of symptoms at a much younger age, engage in more aggressive behaviors, and develop serious delinquency, resulting in greater dysfunction in adulthood. Impulsivity is the component of ADHD that usually increases over time, generating additional symptoms of conduct disorder. By inhibiting self-regulation processes, the ADHD patient is constantly irritable and is involved in impulsive behaviors based on extreme sensation-seeking. They change jobs frequently and start new relationships impulsively. The tendency toward destructive behaviors and the continuous need to be continuously stimulated determine an increased predisposition for these patients to be involved in car accidents, delinquent acts, unbalanced family relationships, and early parenthood (unintended pregnancies before the age of 18) [8].

In light of these findings, the presence of comorbid conduct disorder should be examined as a mediator in the relationship between childhood ADHD and early pregnancy.

Behavioral under-control is associated not only with the persistence of ADHD symptoms but also with drugs/alcohol abuse. Unwanted pregnancies at a young age are correlated with a deviant pattern of behaviors, such as delinquency and the abuse of psychoactive substances. Comorbidity rates among people with ADHD who have a history of addiction or dependence on psychostimulants range from 25% to 55%, according to studies that have examined the existence of this condition [15]. Taking into account the prevalence rate in the overall population (15–18%), we can conclude that having ADHD doubles a person’s risk of developing psychoactive substance misuse [15]. These individuals have a higher propensity to begin abusing drugs at a young age, using many drugs at once, and remaining in treatment programs that combine medication and psychotherapy for longer periods of time. When compared to the control group, adolescents with ADHD start abusing drugs and alcohol at a younger age, and this trend continues until around the age of 40. This fact is possibly associated with the loss of hope for symptom relief. According to specialized studies, there is no predilection for a certain psychoactive substance, and adults with ADHD use all types of drugs. In our current psychiatric evaluation of adolescent girls who chronically use psychoactive substances, we detected repeated sexual behaviors with unknown partners in order to obtain drugs. This demonstrates another factor that predicts sexual risk-taking, early or unwanted pregnancies, and other psychosocial concerns.

It is known that ADHD is a neurodevelopmental disorder with genetic determinism but with a strong educational influence. The pattern of educational attitude of the parents of children with ADHD can influence premature sexual activity. A meta-analysis that assessed the relationship between the parental monitoring of adolescents’ social activities and relationships and early sexual behaviors concluded that positive sexual decision making was influenced by supportive and constant parental monitoring [16]. The parental enforcement of rules and firm guidance were correlated with delaying starting sexual activities, the use of condoms and contraceptives, and positive outcomes of long-term health trajectories. Neglectful parenting, insufficient family support, and unstructured parental bonding increase the risk of externalizing problems in teenagers. Thus, low quality of an educational family system can be another explanatory mechanism for risky sexual behaviors and early pregnancies. 

In the case of adolescents with ADHD, family life is also affected given the increased hereditary risk of this disorder. Adults with ADHD are at risk of having a negatively impacted parent–child relationship [17]. Financial resources are often at a low level due to lower academic performance compared to people with the same cognitive abilities. Many adults with ADHD experience feelings of loneliness and isolation due to social difficulties and shame caused by failures, and their mental and physical well-being is at a lower level, even in the presence of an increased IQ. Lower educational achievements, regardless their intellectual potential; failure in finish their educational programs; unstable job positions as a result of experiencing continuous boredom; and conflicts with colleagues determines poor family functioning and failure to reach their full potential. The bidirectional relationship between school dropout/academic failure and involvement in risky sexual behaviors and early pregnancies represents a vast future research topic.

The above-mentioned mediating factors have been investigated in recent studies, and these consistent results across diverse population cohorts demonstrate the link between ADHD symptoms and risk-taking behavior. A summary of these studies and their conclusions regarding the mediating factors in the occurrence of unplanned pregnancies and risky sexual behaviors is listed in Table 1:

## 4. Birth Outcomes Related to Teenage Pregnancies

Teenage pregnancy represents a complex interplay of health, social, and policy factors, presenting unique challenges for adolescent mothers and their children [26]. Comprehensive research on these various risks, as well as evidence-based mitigation techniques, appear to be lacking at the moment. In addition to highlighting evidence-based strategies to lessen their impact, this narrative synthesis examines these complex risks. Wong et al. found that teenage mothers are at serious risk for obstetric complications [27]. Low birth weight (LBW) and preterm birth (PTB) are among the complications that adolescents are biologically more likely to experience. Lower Apgar scores and other compromised health indicators are common in newborns born to teenage mothers, highlighting the importance of effective prenatal care.

These results are consistent with the broader literature, indicating that detrimental neonatal outcomes are largely caused by biological immaturity and limited access to prenatal care. Teenage mothers are more likely to undergo obstructed labor and have limited pelvic development, which increases the likelihood of complications during childbirth. The health of the mother and the baby is at risk due to conditions like anemia and preeclampsia, which are more prevalent in teenage mothers [28].

Education on dietary and health requirements during pregnancy as well as focused prenatal care are necessary to address these issues. The rate of adolescent births is a crucial measure of reproductive health on a global scale. In 2000, there were 64 out of every 1000 births among women aged 15 to 19 worldwide; by 2023, that number had dropped to 41 out of every 1000, according to the World Health Organization (WHO) [29]. However, regional disparities persist. High rates of adolescent births frequently point to structural problems like lack of access to reproductive health and educational resources, socioeconomic disparities, and cultural norms that encourage early marriage and parenthood. Complex behaviors that represent the core of ADHD impulsivity can be correlated to the differences and increase in teenage pregnancies in some regions (“In the WHO African Region, estimations were 97 per 1000 adolescent in 2023 compared to 13.1 per 1000 adolescent girls in the European Region”) [29].

Adolescent pregnancies are both a cause and an effect of socioeconomic disadvantage from a social standpoint. According to Laurenzi et al., adolescents from low-income neighborhoods usually face structural obstacles to healthcare and education [30]. Dropping out of school frequently occurs before becoming pregnant, which restricts opportunities and prolongs poverty cycles. The vulnerability of teenage mothers is increased by mental health issues, such as elevated rates of ADHD. Often, social stigma and insufficient social support networks exacerbate these psychological burdens.

This group of patients may be discouraged from seeking necessary services due to the stigma attached to adolescent motherhood and the impaired inhibition processes that are typical of ADHD teenagers. SmithBattle emphasizes the value of community-based initiatives that normalize adolescent motherhood, build resilience, and offer strong support networks [31]. By increasing access to healthcare, education, and other support networks, these partnerships play a critical role in mitigating the long-term socioeconomic effects of adolescent pregnancy. To address these disparities, interventions that take into account the unique needs and challenges that these patient categories face must be culturally sensitive. Community-based health education initiatives that are culturally sensitive have shown promise in reaching at-risk groups and promoting the use of contraceptives [32].

## 5. Proposed Measures to Address the Medical and Social Determinants of Health

These interrelated hazards underscore the pressing necessity for coordinated measures that concurrently address the medical and social determinants of health. For example, increasing prenatal care access can lower obstetric risks and psychosocial programs can improve mental health and facilitate reintegration into school. Policymakers, medical professionals, and community organizations must work together to break the cycle of disadvantage and ensure that adolescent mothers and their children have healthier futures.

School-based health programs that provide comprehensive sex education, free contraceptives, and counseling services are examples of promising interventions. Furthermore, mentorship programs that pair adolescent mothers with exemplary role models have proven successful in fostering healthy parenting habits and lessening feelings of loneliness. The primary aim of interventions should be facilitating access to monitoring and counseling services that address the prevention of recurrent pregnancies, which worsen the health and financial difficulties faced by adolescent mothers. Additionally, the revolution in digital health opens up new avenues for intervention. Teenage mothers can now receive critical health information, remote consultations, and continuous support through mobile health apps and telemedicine, which effectively overcome logistical and geographic obstacles to healthcare access.

Mental health specialists can adopt evidence-based approaches to prevent teenage pregnancy and promote sexual health. In Table 2, we include some key recommendations [33,34,35]:

Given its widespread implications, teenage pregnancy represents a pressing public health concern that requires a collective response. Effective interventions must target both obstetric risks through improved prenatal care and social determinants by enhancing education, mental health support, and access to contraception. Healthcare providers must collaborate to design programs that acknowledge the unique experiences of adolescent mothers, ultimately disrupting cycles of poverty and poor health.

## 6. Limitations

Our review has several limitations. Firstly, pertinent research published in other databases or in languages other than English may not have been included in our PubMed-only, English-language literature search. Furthermore, relevant research that utilized different terminologies or concentrated on related but different issues would have been excluded as a result of the usage of certain keywords. We believe that multicentric prospective studies are crucial for developing uniform and useful clinical guidelines, because the information provided may be biased by our local hospital resources and our national clinical guidelines.

## 7. Conclusions 

The mechanisms underlying the impulsive sexual behaviors to which girls diagnosed with ADHD are exposed are far from being clarified. The negative associations for school achievements, the presence of comorbidities, overly flexible parenting styles (e.g., excessively adaptable parenting approaches or inconsistency in the application of intrafamilial rules), and the predisposition toward drug use are only some of the factors that mediate the relationship between the persistence of ADHD symptoms in young girls and unwanted pregnancies. There is evidence to indicate the negative effects of an unwanted pregnancy on offspring development and adjustment. Further research is needed in order to monitor and detail these negative effects on different developmental stages but also on the potential physical/somatic diseases of these children as a result of teenage pregnancies.

The causal pathways that can explain risky sexual behaviors and unplanned pregnancies in ADHD girls take shape in this research, but there is still a gap in identifying long-term effects and health outcomes in this category of patients. 

## Figures and Tables

**Table 1:** Mediating factors in the occurrence of unplanned pregnancies or risky sexual behaviors.

Study	Study Population	Factors	*p* Value *	Ref.
E. B. Owens et al. (2019)	140 girls with ADHD 88 controls	Impulsivity	0.149	[11]
		Hyperactivity	0.001	
		Conduct problems	<0.001	
		Substance use	0.608	
		Academic achievement	<0.001	
		Risky sex	<0.001	
J. Isaksson et al. (2017)	537 participants	Inattention symptoms	<0.001	[18]
		Hyperactivity/impulsivity symptoms	<0.001	
		Conduct problems	<0.001	
		Substance use	0.002	
		Perception of risk	<0.001	
		Parental involvment	0.05	
K. Flory et al. (2006)	175 ADHD patients 111 controls	Oppositional defiant disorder	<0.05	[19]
		Conduct disorder	<0.05	
P. J. Bachanas et al. (2002)	158 participants	Depression	<0.01	[20]
		Conduct problems	<0.01	
		Substance use	<0.01	
		Social support	<0.01	
D. E. Sarver et al. (2014)	115 participants	Hyperactivity/impulsivity symptoms	<0.001	[21]
		Oppositional defiant disorder	<0.05	
		Conduct disoder	<0.05	
		Substance use	0.02	
E. B. Owens et al. (2017)	140 girls with ADHD 88 controls	Family income	0.002	[8]
		Innatention symptoms	<0.001	
		Hyperactivity/impulsivity symptoms	<0.001	
		Externalizing problems	<0.001	
		Internalizing problems	<0.001	
M. C. Meinzer et al. (2020)	579 ADHD patients 289 controls	Persistent ADHD symptoms	<0.01	[4]
		Delinquency/substance use	<0.01	
		Lower academic performance	<0.01	
		Parenting during adolescence	0.03	
M. H. Hua et al. (2020)	7505 ADHD patients 30,020 controls	Psychiatric comorbidities	<0.001	[22]
		Disruptive behavior disorders	<0.001	
		Alcohol use disorders	<0.001	
		Substance use disorders	<0.001	
G. M. M. Hosain et al. (2012)	462 participants	Sex before 15 years of age	0.008	[23]
		Risky sex partners in lifetime	<0.0001	
		Substance use	0.007	
S. Seppa et al. (2023)	9432 participants	Oppositional defiant disorder	<0.001	[24]
		Conduct disoder	<0.001	
		Educational underachievement	<0.001	
K. Skoglund et al. (2019)	6410 ADHD patients 377,693 controls	Comorbid psychiatric disorders	<0.01	[25]

* *p* value < 0.05 reflects a statistically significant correlation of the studied mediating factors in the occurrence of unplanned pregnancies or risky sexual behaviors in adolescent ADHD patients.

**Table 2:** Proposed interventions for addressing medical and social determinants of health.

Comprehensive Sex Education	- Delivering age-appropriate, evidence-based sex education that encompasses contraception, healthy relationships, and the consequences of early pregnancy. - Research over the past decade demonstrates that comprehensive sex education significantly reduces adolescent birth rates.
Mental Health Screening and Support	- Psychiatrists should screen adolescents for ADHD and other risk factors, acknowledging the strong links between mental health challenges and teenage pregnancies. - Promoting mental health resilience through counseling and support can empower teenagers to make informed choices regarding their sexual health.
Facilitating Access to Care	- Psychiatrists can refer adolescents to local resources, including family planning clinics, support groups, and educational workshops, which can provide access to contraception and reproductive healthcare services.
Family Involvement	- Engaging families in discussions about sexual health and pregnancy prevention can create a supportive environment for adolescents. - Specialists can assist parents in establishing open lines of communication and addressing cultural or religious concerns.
Evidence-Based Digital Platforms	- Digital tools, such as mobile health applications, can deliver discreet and accessible sexual health and contraception information to adolescents.
Advocacy	- Mental health specialists should advocate for policies that promote adolescent health, including increased funding for sex education programs and improved access to contraception. - Collaboration with community organizations and schools is vital for implementing these policies.

## Data Availability

Not applicable.
